# Effect of Oxybutynin on Detrusor Leak Point Pressure in Spinal Cord Injury Patients With Neurogenic Detrusor Overactivity: A Subgroup Analysis

**DOI:** 10.7759/cureus.82865

**Published:** 2025-04-23

**Authors:** Vineet Bharti, Mrinal Joshi, Neha Saini

**Affiliations:** 1 Physical Medicine and Rehabilitation, Employees State Insurance Corporation (ESIC) Medical College and Hospital, Faridabad, IND; 2 Physical Medicine and Rehabilitation, Sawai Man Singh (SMS) Medical College, Jaipur, IND; 3 Microbiology, Vardhman Mahavir Medical College and Safdarjung Hospital, Faridabad, IND

**Keywords:** bladder compliance, cervical injury, chronic injury, detrusor hyperreflexia, detrusor leak point pressure, neurogenic detrusor overactivity, oxybutynin, spinal cord injury, subgroup analysis, urodynamics

## Abstract

Following spinal cord injury, neurogenic detrusor overactivity elevates detrusor leak point pressure, endangering renal function. This research examined oxybutynin's effects on detrusor leak point pressure and urodynamic parameters in 30 individuals with spinal cord injury and neurogenic detrusor overactivity, emphasizing variations across subgroups. Patients were given 5 mg of oxybutynin each day for a week, with urodynamic assessments conducted pre- and post-treatment. Decreases in detrusor leak point pressure were noted, especially among those with detrusor hyperreflexia combined with detrusor sphincter dyssynergia, cervical-level injuries, and injuries exceeding six months. Improvements in bladder compliance and capacity occurred universally, yet detrusor leak point pressure reductions differed by subgroup. Oxybutynin proves useful in reducing detrusor leak point pressure in vulnerable spinal cord injury patients, promoting its application in customized neurogenic detrusor overactivity care.

## Introduction

Spinal cord injury (SCI) is a profound condition often caused by traumatic incidents, such as motor vehicle crashes or falls, or non-traumatic factors such as vascular disorders or infections [1]. Globally, traumatic SCI incidence spans 3.6 to 195.4 cases per million people annually, with developing countries averaging 25.5 cases per million [2]. Over the past seven decades, enhanced medical interventions, particularly in urinary tract care, have significantly extended survival, reducing mortality from urological issues [3,4]. Neurogenic bladder dysfunction affects over 90% of SCI patients, heightening risks of urinary tract infections (UTIs), kidney damage, and bladder stones [5,6].

Urodynamic assessments have revolutionized the management of bladder dysfunction post-SCI, revealing how neurogenic detrusor overactivity (NDO), characterized by involuntary detrusor contractions during filling, increases detrusor leak point pressure (DLPP) [7]. A DLPP exceeding 40 cm H₂O threatens upper urinary tract health, potentially causing vesico-ureteric reflux (VUR) and renal impairment [8]. Effective treatment focuses on preserving kidney function and enhancing the quality of life by maintaining low bladder storage pressures and improving compliance [9].

Oxybutynin, an antimuscarinic medication with muscle-relaxant properties, is a primary therapy for NDO [10]. Its metabolite, N-desethyl oxybutynin, suppresses unintended bladder contractions by inhibiting muscarinic receptors [11]. Combined with clean intermittent catheterization (CIC), it achieves renal protection and continence in over 90% of patients [12]. Despite its global use, evidence about its effectiveness in specific contexts, such as India, is limited. This study evaluates oxybutynin's impact on DLPP, bladder compliance, and capacity in SCI patients with NDO, analyzing variations across injury and clinical subgroups.

## Materials and methods

Study design and setting

This prospective, non-randomized interventional study was conducted at the Department of Physical Medicine and Rehabilitation, Sawai Man Singh (SMS) Medical College and Hospitals, Jaipur, India, from July 2015 to September 2016.

Participants

Thirty SCI patients with urodynamically confirmed NDO were enrolled via consecutive sampling. Inclusion criteria included age over 18 years, SCI duration exceeding three months (post-spinal shock), American Spinal Injury Association (ASIA) classification A-D, DLPP above 40 cm H₂O, and informed consent. Exclusion criteria encompassed medications altering bladder function, conditions complicating urodynamic tests, detrusor areflexia, active UTIs, or oxybutynin contraindications (e.g., glaucoma). The sample size was calculated as 26, based on a 96.3 mL mean difference in cystometric capacity (SD 167.71), with an alpha of 0.05 and 80% power, increased to 30 to account for 10% attrition.

Intervention

Participants were given 5 mg of oral oxybutynin daily for seven days. Those with UTIs (symptomatic or asymptomatic) received seven days of antibiotics, guided by urine culture sensitivity, before urodynamic assessments.

Urodynamic evaluation

Pre- and post-treatment urodynamic tests were performed using a Laborie urodynamic system (Laborie Medical Technologies, Portsmouth, New Hampshire, USA). The protocol involved cystometry (filling and voiding phases) and perineal electromyography (EMG) in a supine right oblique position. Intravesical pressure was measured via a 7F double-lumen catheter, and abdominal pressure via a 10F rectal catheter. Bladder filling occurred at 10 mL/minute with 2% xylocaine jelly lubrication. Measured parameters included DLPP, bladder compliance (mL/cm H₂O), and capacity (mL).

Statistical analysis

Paired t-tests evaluated changes in urodynamic outcomes, with subgroup analyses based on detrusor hyperreflexia (DH) with/without detrusor sphincter dyssynergia (DSD), injury level (cervical vs. dorsal/lumbar), and injury duration (>6 months vs. ≤6 months). Chi-square tests assessed associations between neurological level/ASIA grade and bladder patterns. Significance was defined as P < 0.05.

## Results

Participant characteristics

Of the 30 participants, 93.3% were male, 53.3% were 30 years or younger, and 63.3% had formal education. Most injuries (56.7%) were less than six months old, primarily caused by falls (46.7%) or road traffic accidents (43.3%). Cervical and lower dorsal injuries comprised 80% of cases, with 86.7% classified as ASIA A. Bladder patterns included 73.3% with DH and DSD, 23.3% with DH alone, and 86.7% lacked a bulbocavernosus reflex.

Urodynamic outcomes

Overall, DLPP decreased from 56.19 ± 35.36 to 42.39 ± 46.11 cm H₂O (P=0.036, t=1.988), compliance improved from 6.11 ± 10.59 to 17.95 ± 33.16 mL/cm H₂O (P=0.023, t=-2.393), and capacity rose from 165.3 ± 163.8 to 229.7 ± 184.5 mL (P<0.001, t=-3.872).

Subgroup analysis

Subgroup analysis revealed varied responses to oxybutynin treatment across different patient characteristics, as detailed in Tables 1, 2. Significant reductions in DLPP were observed in patients with DH combined with DSD, those with cervical injuries, and individuals with chronic injuries exceeding six months. In contrast, patients with DH alone or dorsal/lumbar injuries showed less pronounced changes in DLPP. Bladder capacity increased significantly across most subgroups, particularly in those with DH and DSD, cervical injuries, and chronic injuries, with notable improvements in compliance for the DH with DSD and cervical injury subgroups.

**Table 1:** Changes in Detrusor Leak Point Pressure (DLPP) Across Subgroups DH: Detrusor Hyperreflexia; DSD: Detrusor Sphincter Dyssynergia; DLPP: Detrusor Leak Point Pressure

Subgroup	N	Pre-DLPP (cm H₂O)	Post-DLPP (cm H₂O)	P-value	t-value
DH with DSD	22	55.3 ± 37.17	38.96 ± 28.02	0.017	2.62
DH Alone	8	58.64 ± 32.01	51.8 ± 79.46	0.745	0.33
Cervical	12	47.77 ± 25.87	33.18 ± 27.55	0.005	3.52
Dorsal/Lumbar	18	61.81 ± 40.2	48.52 ± 55.1	0.259	1.17
>6 Months	13	53.43 ± 42.58	33.06 ± 27.99	0.008	3.00
≤6 Months	17	58.51 ± 29.92	49.52 ± 57.16	0.446	0.78

**Table 2:** Changes in Bladder Capacity Across Subgroups DH: Detrusor Hyperreflexia; DSD: Detrusor Sphincter Dyssynergia

Subgroup	N	Pre-Capacity (mL)	Post-Capacity (mL)	P-value	t-value
DH with DSD	22	152.3 ± 170.1	240.8 ± 195.2	<0.001	4.89
DH Alone	8	198.7 ± 160.4	203.5 ± 175.9	0.842	0.21
Cervical	12	175.9 ± 180.2	235.4 ± 205.3	0.002	3.85
Dorsal/Lumbar	18	158.6 ± 160.3	225.8 ± 183.7	0.004	3.22
>6 Months	13	170.2 ± 175.3	245.6 ± 200.1	<0.001	4.33
≤6 Months	17	162.1 ± 163.8	216.9 ± 184.5	0.009	2.91

## Discussion

Oxybutynin at 5 mg daily for one week reduced DLPP in SCI patients with NDO, with significant effects in those with DH and DSD (P = 0.017), cervical injuries (P = 0.005), and chronic injuries over six months (P = 0.008). This aligns with its mechanism, antagonizing muscarinic receptors and relaxing the detrusor muscle, effectively curbing overactivity [10,13]. The notable DLPP reduction in the DH with DSD subgroup underscores its efficacy in managing combined detrusor sphincter dysfunction, consistent with prior findings [14]. Cervical injury patients showed a strong response, likely due to higher baseline pressures from upper spinal lesions, as noted in studies linking injury level to bladder dynamics [15].

Chronic injuries (>6 months) exhibited robust improvements, possibly reflecting stabilized neural changes that enhance treatment response [16]. Bladder compliance improved significantly in the DH with DSD and cervical subgroups (P < 0.001 and P = 0.019, respectively), while capacity increased universally, with the greatest gains in DH with DSD and chronic cases (P < 0.001) [17]. These results echo findings from Kim et al. (1997), who reported better compliance and lower DLPP with reduced hydronephrosis in SCI patients on oxybutynin [12]. Similarly, Di Stasi et al. (2001) observed significant urodynamic improvements with oxybutynin in neurogenic bladder patients, reinforcing its therapeutic role [18].

The lack of significant DLPP changes in DH alone, dorsal/lumbar injuries, and acute cases may stem from smaller subgroup sizes or variable baseline pressures, a pattern seen in Madhuvrata et al.'s (2012) review of anticholinergics for NDO [15]. This study's emphasis on urodynamic-guided therapy aligns with Linsenmeyer et al. (2013), who advocate regular urodynamic monitoring to optimize bladder management and prevent renal damage [16]. Panicker et al. (2015) further support integrating clinical and urodynamic data for tailored NDO treatment [7]. The absence of correlation between neurological level or ASIA grade and bladder pattern (P = 0.800 and P = 0.599) supports Aggarwal and Joshi (2015), who noted discrepancies between somatic findings and urodynamic outcomes, advocating routine urodynamic evaluations [13].

The limitations of this study include a modest sample size (n = 30), short treatment duration (one week), and a non-randomized design, which may introduce bias. The effectiveness of bladder emptying methods (e.g., CIC) could not be fully assessed due to limited subgroup numbers, though prior studies confirm CIC's safety in SCI management [19]. Future research should involve larger, randomized trials comparing oxybutynin with newer agents like tolterodine or solifenacin, especially in resource-constrained settings where its low cost is advantageous [20].

## Conclusions

Oxybutynin at 5 mg daily effectively lowers DLPP and enhances bladder compliance and capacity in SCI patients with NDO, with superior outcomes in DH with DSD, cervical injuries, and chronic cases (>6 months). These results support its use for personalized bladder management, particularly in high-risk patients, and highlight its value due to broad efficacy and affordability. Larger studies comparing it to modern alternatives could refine its application, but its cost-effectiveness makes it a practical choice for NDO treatment in regions like India.
